# Nutritional Status and Its Association With Radiation-Induced Oral Mucositis in Patients With Nasopharyngeal Carcinoma During Radiotherapy: A Prospective Study

**DOI:** 10.3389/fonc.2020.594687

**Published:** 2020-11-06

**Authors:** Zekai Shu, Ziyi Zeng, Bingqi Yu, Shuang Huang, Yonghong Hua, Ting Jin, Changjuan Tao, Lei Wang, Caineng Cao, Zumin Xu, Qifeng Jin, Feng Jiang, Xinglai Feng, Yongfeng Piao, Jing Huang, Jia Chen, Wei Shen, Xiaozhong Chen, Hui Wu, Xiushen Wang, Rongliang Qiu, Lixia Lu, Yuanyuan Chen

**Affiliations:** ^1^ Department of Radiation Oncology, Cancer Hospital of the University of Chinese Academy of Sciences (Zhejiang Cancer Hospital), Hangzhou, China; ^2^ Institute of Cancer and Basic Medicine (IBMC), Chinese Academy of Sciences, Hangzhou, China; ^3^ The Second Clinical Medical College, Zhejiang Chinese Medical University, Hangzhou, China; ^4^ State Key Laboratory of Oncology in South China, Collaborative Innovation Center for Cancer Medicine, Guangdong Key Laboratory of Nasopharyngeal Carcinoma Diagnosis and Therapy, Sun Yat-sen University Cancer Center, Guangzhou, China; ^5^ Department of Radiation Oncology, Sun Yat-sen University Cancer Center, Guangzhou, China; ^6^ Department of Oncology, Zhejiang Hospital, Hangzhou, China; ^7^ Cancer Center, Affiliated Hospital of Guangdong Medical University, Zhanjiang, China; ^8^ Department of Radiation Oncology, The Affiliated Huaian No.1 People’s Hospital of Nanjing Medical University, Huaian, China; ^9^ Hangzhou YITU Healthcare Technology Co., Ltd, Hangzhou, China; ^10^ Department of Radiation Oncology, Affiliated Cancer Hospital of Zhengzhou University, Zhengzhou, China; ^11^ Chinese Society of Nutritional Oncology, CSNO, Tianjin, China

**Keywords:** radiation–induced oral mucositis, radiotherapy, nutritional status, nasopharyngeal carcinoma (NPC), head and neck cancer

## Abstract

**Background and Aims:**

Malnutrition is a concern in patients with nasopharyngeal carcinoma (NPC) during chemoradiotherapy (CRT)/radiotherapy (RT), which is considered to be related with radiation–induced oral mucositis (ROM). The study aimed to evaluate the nutritional status of NPC patients during RT and investigate its association with ROM.

**Methods:**

A prospective study was conducted in NPC patients. Patients were divided into three subgroups (mild, moderate, and severe groups) based on the duration of severe ROM (≥ grade 3). Body weight, body mass index (BMI), albumin, prealbumin, NRS2002, and ROM grade were assessed on a weekly basis before and during CRT/RT. The statistical analysis was performed in the overall group and between three subgroups.

**Results:**

A total of 176 patients were included. In the overall group, body weight and BMI kept decreasing since week 1 of RT, and NRS2002 score and ROM grade increased (p < 0.001). NRS2002 score and prealbumin levels were significantly different between each subgroup (p ≤ 0.046). Significant differences were observed in the proportion of patients receiving enteral nutrition, duration of parenteral nutrition, and total calories provided by nutritional support among three subgroups (p = 0.045–0.001).

**Conclusions:**

Malnutrition occurred early in NPC patients and worsened continuously during RT. ROM was strongly associated with nutritional status. Nutritional support should be provided at the start of RT, especially in patients at high-risk of severe ROM.

## Introduction

Nasopharyngeal carcinoma (NPC), an epithelial cell cancer in the nasopharynx, is a rare malignancy (1, 2). Even though the annual global incidence is 1.2 per 100,000 individuals, NPC represents a health burden in Southern China, Southeastern Asia, and Southern Africa with more than 70% of new cases distributed in these areas (1). According to the guidelines established by the National Comprehensive Cancer Network (NCCN), the standard treatment consists of CRT with/without neoadjuvant chemotherapy, which is dependent on the cancer stage and physical condition of the patient (2, 3). With novel RT technology, intensity-modulated radiotherapy (IMRT), and helical tomotherapy (TOMO), CRT treatment provides 80% of 5–year survival rate and 90% of 3–year locoregional control rate (4, 5).

Although the prognosis of NPC is good, certain acute side effects of RT may affect the course of treatment, including xerostomia, skin reactions, hearing loss, pharyngitis, vomiting, and radiation-induced oral mucositis (ROM) (5–7). ROM, which may lead to serious consequences (8, 9), occurs in over 90% of patients with head and neck cancer (HNC) and almost all NPC patients, 34% to 66% of whom develop severe ROM (≥ grade 3) (8, 10, 11). In severe ROM, patients experience ulceration, necrosis, severe oral pain, and malnutrition due to difficulties in food intake (10, 12, 13). In mild ROM (≤ grade 2), 38% of patients still experience difficulties with food intake (8).

The nutritional status of patients will deteriorate, which can lead to severe weight loss, poor physical condition, and treatment interruption (14–16). However, there is no direct evidence on the extent of ROM’s impact on nutritional status. This prospective study investigated the nutritional status of NPC patients during RT and its association with ROM. We assessed body weight, BMI, serum albumin, prealbumin, NRS2002 score, and ROM grade on a weekly basis according CTCAE 4.0 (Grade 1-5, **Supplemental Table 1**) (17). Data of this study were acquired from a multi–centric randomized controlled trial (RCT; NCT03720340).

## Material and Methods

### Study Population

This study was conducted in five medical centers. The inclusion criteria: 1) NPC patients with confirmed pathogenesis; 2) cancer stages I–IVB according to the 8th version of the American Joint Committee on Cancer; 3) patients between the ages of 18 and 75 years; 4) performance status of 0 or 1 based on the Eastern Cooperative Oncology Group; 5) no bone marrow, renal, hepatic disorders; 6) patients willing to participate in the study and sign an informed consent. The exclusion criteria were the following, 1) treatment with palliative intent; 2) patients with previous malignancy; 3) pregnancy or lactation; 4) patients who underwent radiotherapy, chemotherapy, or surgery (except biopsy operation) for primary tumors or nodes; 5) patients of oral mucositis or senile dry stomatitis before treatment; or 6) presence of severe comorbidities. 7) chemotherapy with fluorouracil drugs; allergies to recombinant human interleukin-11.

### Treatment Plan

IMRT or TOMO plan was implemented before RT as previously reported (18, 19). Radiation was delivered five times a week from Monday through Friday for six to seven weeks (an average of six and a half weeks). Platinum-based drugs were used in neoadjuvant and concurrent chemotherapy. The most commonly used regimen was 3 cycles of neoadjuvant chemotherapy and 0 to 2 cycles of concurrent chemotherapy. Some patients received 3 to 8 cycles of concurrent nimotuzumab (200 mg/week; 7 cycles for most patients).

### Nutrition Supplement

As data of this study were acquired from an RCT, there were no standard guidelines or procedures for nutritional support. Nutritional support (commercial products) was provided based on the nutritional status of the patient and consisted of an enteral nutrition supplement (oral nutrition administered and enteral tube feeding) and a parenteral nutrition supplement.

### Data Collection

Clinical data were collected before (T0) and at the end of each week during RT (T1–T6). Clinical data included patient characteristics (age, sex, Barthel index score, tumor stage, smoking history, alcohol consumption history, and treatment plan, etc), nutritional factors (body weight, BMI, serum albumin, and prealbumin), nutritional support (number of patients receiving enteral and parenteral nutrition, duration of parenteral nutrition, and total calories of enteral and parenteral nutrition supplements).

A trained clinical research coordinator (CRC) evaluated the nutrition status of every patient according to NRS2002 before and during RT (20). Additionally, ROM grade was assessed according to CTCAE 4.0 (Grade 1-5, **Supplemental Table 1**) (17).

### Statistical Analysis

We calculated mean and standard division (SD) for continuous variables and frequency for categorical variables. We used paired Student’s t-test to compare differences between T0 and T1–T6 in NRS2002 score, ROM grade, body weight ratio (BWR, ratio of body weight at T1-T6 to T0), BMI, serum albumin, and prealbumin. Variance analysis (ANOVA) was used to assess differences in NRS2002 score, ROM grade, BWR, BMI, serum albumin, prealbumin, duration of parenteral nutrition supplement, starting time of enteral and parenteral nutrition supplement, and total calories of enteral and parenteral nutrition. Chi-square test was used in the analysis of the proportion of patients receiving general nutrition support, enteral nutrition, and parenteral nutrition. Statistically significance was set at p < 0.05. SPSS software version 24.0 (IBM Corp., Armonk, NY, USA) was used for all statistical analyses.

## Results

### Patient Characteristics

The RCT involved 272 patients up to Jan 2020. In this study, 176 patients with comprehensive data were included. The basic clinical characteristics are presented in **Table 1**. There were 122 (69.3%) males and 54 (30.7%) female patients with a median age of 51 years. Body weight and BMI before RT were 65.99 ± 11.00 kg and 23.98 ± 3.23, respectively. A total of 167 (94.9%) patients received neoadjuvant chemotherapy, 157 (89.2%) patients received concurrent chemotherapy, and 118 (67.0%) received nimotuzumab. The most common radiation technology was IMRT (69.9%), followed by TOMO (30.1%).

**Table 1:** Patient characteristics.

Characteristics		
Age, median (range)	years	51 (18–73)
Sex ratio, mean ± SD	M/F	122/54
Body weight, mean ± SD	kg	65.99 ± 11.00
BMI, mean ± SD		23.98 ± 3.23
Barthel index, median (range)		90 (85-100)
Smoking history, n (%)	Yes	76 (43.2)
	No	100 (56.8)
Drinking history, n (%)	Yes	50 (28.4)
	No	126 (71.6)
T stage, n (%)	T1-2	35 (19.9)
	T3-4	141 (80.1)
N stage, n (%)	N0-2	140 (79.5)
	N3	36 (20.5)
Neo-chemotherapy, n (%)	Yes	167 (94.9)
	No	9 (5.1)
Concurrent chemotherapy, n (%)	Yes	157 (89.2)
	No	19 (10.8)
Radiation technology, n (%)	IMRT	123 (69.9)
	TOMO	53 (30.1)
Nimotuzumab, n (%)	Yes	118 (67.0)
	No	58 (33.0)

BMI, body mass index.

### Nutritional Status and Oral Condition in the Overall Group During RT

**Figure 1:**
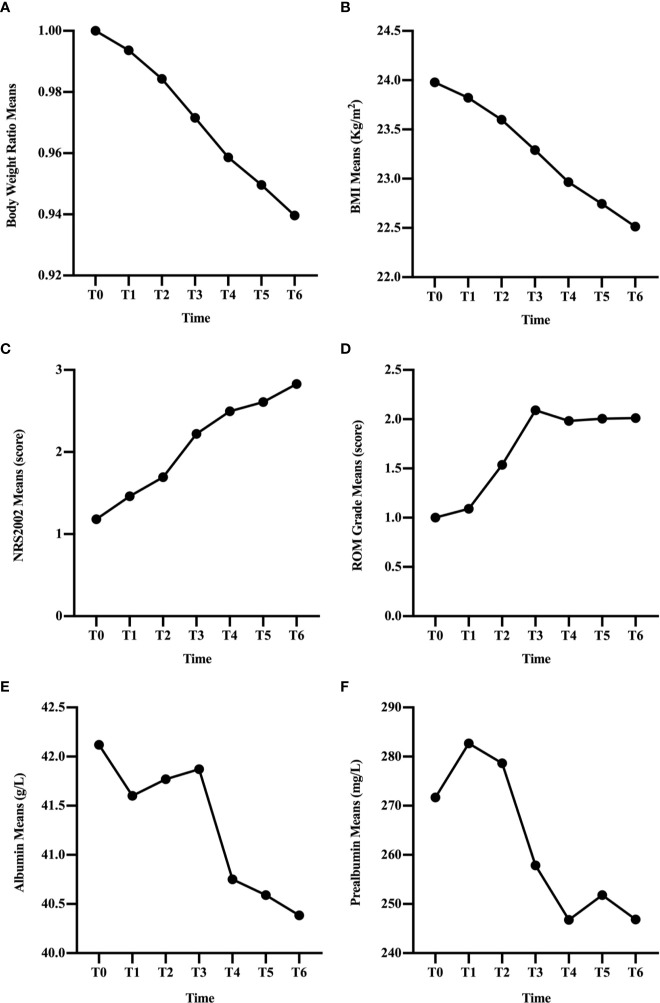
Changes in clinical factors, BWR **(A)**, BMI **(B)**, NRS2002 score **(C)**, ROM grade **(D)**, albumin **(E)**, and prealbumin **(F)**, of all patients during CRT/RT treatment.

In the overall group, BWR and BMI decreased since the beginning of RT (**Figures 1A, B**) and the differences remained significant since T1 (p < 0.001; **Table 2**). NRS2002 score increased from the start of treatment (**Figure 1C**), and the differences in the scores were statistically significant from T1 (p < 0.001; **Table 2**). A similar trend was obtained in ROM grade, which reached its maximum value at T3 and plateaued thereafter (**Figure 1D**; **Table 2**).

**Table 2:** Changes in clinical factors of all patients during chemoradiotherapy (CRT)/radiotherapy (RT) treatment and comparison between T0 and T1–T6 in NRS2002 score, ROM grade, BWR, BMI, albumin, and prealbumin.

Items, mean ± SD	T0	T1	T2	T3	T4	T5	T6
NRS2002 score	1.18 ± 0.58	1.46 ± 0.79	1.69 ± 0.92	2.22 ± 1.09	2.50 ± 1.14	2.61 ± 1.11	2.83 ± 1.09
*p*-value		**<0.001**	**<0.001**	**<0.001**	**<0.001**	**<0.001**	**<0.001**
ROM Grade	1.00 ± 0.00	1.09 ± 0.29	1.54 ± 0.64	2.09 ± 0.75	1.98 ± 0.76	2.01 ± 0.80	2.01 ± 0.81
*p*-value		**<0.001**	**<0.001**	**<0.001**	**<0.001**	**<0.001**	**<0.001**
BWR	1.00 ± 0.00	0.994 ± 0.017	0.984 ± 0.021	0.972 ± 0.026	0.959 ± 0.028	0.950 ± 0.033	0.940 ± 0.036
*p*-value		**<0.001**	**<0.001**	**<0.001**	**<0.001**	**<0.001**	**<0.001**
BMI	23.98 ± 3.23	23.82 ± 3.23	23.60 ± 3.20	23.29 ± 3.16	22.97 ± 3.06	22.75 ± 3.05	22.51 ± 3.03
*p*-value		**<0.001**	**<0.001**	**<0.001**	**<0.001**	**<0.001**	**<0.001**
Albumin (g/L)	42.10 ± 3.72	41.61 ± 3.68	41.82 ± 3.89	41.96 ± 3.62	40.83 ± 4.63	40.63 ± 4.21	40.37 ± 4.99
*p*-value		0.053	0.347	0.467	**<0.001**	**<0.001**	**<0.001**
Prealbumin (mg/L)	272.22 ± 48.99	282.53 ± 59.15	279.01 ± 61.66	258.19 ± 60.04	246.35 ± 63.04	250.91 ± 66.77	245.33 ± 61.08
*p*-value		**.001**	0.069	**<0.001**	**<0.001**	**<0.001**	**<0.001**

NRS2002, nutrition risk screening 2002; ROM, radiation-induced oral mucositis; BWR, body weight ratio (ratio of body weight at T1-T6 to T0); BMI, body mass index. Paired Student’s t-test was conducted between T0 and T1–T6. Bold values means the p value is less than 0.05.

In general, the change trend of albumin level was downward, but there was a rise at T2 and T3 (**Figure 1E**). And the differences were significant since T4 (p < 0.001; **Table 2**). Prealbumin levels were higher at T1 than at T0 (p < 0.001; **Table 2** and **Figure 1F**) and subsequently decreased with a slight increase at T5. Prealbumin level became significant lower since T3 compared with T0 (p < 0.001, **Table 2**).

### Association Between Nutritional Status and ROM

**Figure 2:**
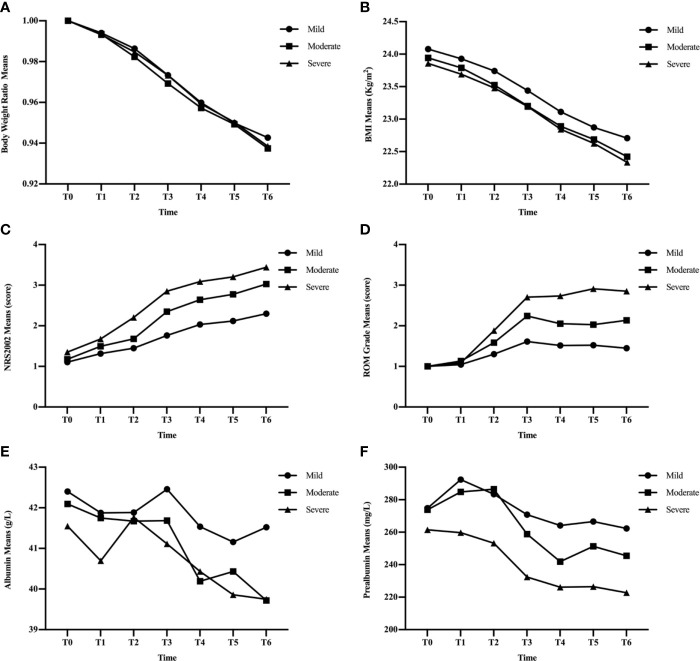
Changes in clinical factors, BWR **(A)**, BMI **(B)**, NRS2002 score **(C)**, ROM grade **(D)**, albumin **(E)**, and prealbumin **(F)**, in mild, moderate, and severe groups during CRT/RT treatment.

To evaluate a possible association between ROM and nutritional status, patients were divided into three subgroups based on the duration of severe ROM (≥ grade 3). Patients without severe ROM were classified as the mild group, and those with severe ROM for 1–2 or ≥ 3 weeks were classified as moderate and severe groups, respectively. There were 67 (38.1%) patients were in the mild group, 75 (42.6%) were in the moderate group, and 34 (19.3%) were in the severe group. **Table 3** and **Figure 2D** showed that ROM grade diverged since T2 between each subgroup (p ≤ 0.020).

**Table 3:** Comparison between mild, moderate, and severe groups in NRS2002 score and ROM grade at T0–T6 during chemoradiotherapy (CRT)/radiotherapy (RT) treatment.

Items, mean ± SD	Mild group	Moderate group	Severe group	*p* (mild vs moderate)	*p* (mild vs severe)	*p* (moderate vs severe)
NRS2002 score						
T0	1.10 ± 0.50	1.17 ± 0.53	1.35 ± 0.77	0.476	**0.041**	0.131
T1	1.31 ± 0.70	1.49 ± 0.81	1.68 ± 0.88	0.175	**0.029**	0.261
T2	1.45 ± 0.80	1.68 ± 0.92	2.21 ± 0.98	0.122	**<0.001**	**0.005**
T3	1.76 ± 1.00	2.35 ± 1.06	2.85 ± 0.96	**0.001**	**<0.001**	**0.017**
T4	2.03 ± 1.08	2.64 ± 1.09	3.09 ± 1.03	**0.001**	**<0.001**	**0.045**
T5	2.12 ± 1.08	2.77 ± 1.06	3.21 ± 0.88	**<0.001**	**<0.001**	**0.045**
T6	2.30 ± 1.14	3.03 ± 0.96	3.44 ± 0.75	**<0.001**	**<0.001**	**0.046**
ROM grade						
T0	1.00 ± 0.00	1.00 ± 0.00	1.00 ± 0.00	–	–	–
T1	1.04 ± 0.21	1.13 ± 0.34	1.09 ± 0.29	0.068	0.473	0.449
T2	1.30 ± 0.46	1.59 ± 0.64	1.88 ± 0.77	**0.006**	**<0.001**	**0.020**
T3	1.61 ± 0.49	2.24 ± 0.75	2.71 ± 0.58	**<0.001**	**<0.001**	**<0.001**
T4	1.52 ± 0.50	2.05 ± 0.71	2.74 ± 0.62	**<0.001**	**<0.001**	**<0.001**
T5	1.52 ± 0.50	2.03 ± 0.80	2.91 ± 0.29	**<0.001**	**<0.001**	**<0.001**
T6	1.45 ± 0.50	2.13 ± 0.79	2.85 ± 0.36	**<0.001**	**<0.001**	**<0.001**

NRS2002, nutrition risk screening 2002; ROM, radiation-induced oral mucositis. Variance analysis was conducted between each group. Bold values means the p value is less than 0.05.

BWR and BMI decreased, while albumin levels increased at certain time points in the three subgroups (**Figures 2A, B, E**). There were no significant differences in BWR, BMI, or albumin between the subgroups at each time point (p > 0.05), except for albumin levels between the mild and moderate groups at T6 (p = 0.035; **Tables 4** and **5**).

**Table 4:** Comparison between mild, moderate, and severe groups in BWR and BMI at T0–T6 during CRT/RT treatment.

Items, mean ± SD	Mild group	Moderate group	Severe group	*p* (mild vs moderate)	*p* (mild vs severe)	*p* (moderate vs severe)
BWR						
T0	1.00 ± 0.00	1.00 ± 0.00	1.00 ± 0.00	–	–	–
T1	0.994 ± 0.020	0.993 ± 0.016	0.993 ± 0.015	0.834	0.803	0.934
T2	0.986 ± 0.025	0.982 ± 0.018	0.985 ± 0.021	0.260	0.708	0.592
T3	0.973 ± 0.028	0.969 ± 0.023	0.973 ± 0.030	0.355	0.984	0.464
T4	0.960 ± 0.028	0.957 ± 0.026	0.959 ± 0.032	0.582	0.922	0.728
T5	0.950 ± 0.033	0.949 ± 0.032	0.950 ± 0.034	0.906	0.958	0.881
T6	0.943 ± 0.036	0.937 ± 0.037	0.938 ± 0.034	0.384	0.580	0.884
BMI						
T0	24.08 ± 3.55	23.94 ± 2.95	23.86 ± 3.26	0.804	0.748	0.900
T1	23.93 ± 3.54	23.79 ± 2.96	23.69 ± 3.22	0.799	0.727	0.883
T2	23.74 ± 3.51	23.52 ± 2.96	23.48 ± 3.14	0.689	0.697	0.943
T3	23.44 ± 3.54	23.30 ± 2.88	23.19 ± 3.07	0.659	0.714	0.988
T4	23.11 ± 3.47	22.89 ± 2.78	22.84 ± 2.87	0.668	0.679	0.942
T5	22.87 ± 3.48	22.69 ± 2.71	22.63 ± 2.93	0.721	0.705	0.925
T6	22.71 ± 3.53	22.42 ± 2.70	22.33 ± 2.70	0.578	0.560	0.888

BWR, body weight ratio (ratio of body weight at T1-T6 to T0); BMI, body mass index. Variance analysis was conducted between each group.

**Table 5:** Comparison between mild, moderate, and severe groups in albumin and prealbumin levels at T0–T6 during chemoradiotherapy (CRT)/radiotherapy (RT) treatment.

Items, mean ± SD	Mild group	Moderate group	Severe group	*p* (mild vs moderate)	*p* (mild vs severe)	*p* (moderate vs severe)
Albumin (g/L)						
T0	42.40 ± 3.30	42.09 ± 4.23	41.55 ± 3.20	0.626	0.298	0.500
T1	41.87 ± 3.40	41.75 ± 3.93	40.69 ± 3.47	0.836	0.135	0.174
T2	41.88 ± 3.19	41.67 ± 4.42	41.76 ± 3.82	0.748	0.884	0.911
T3	42.46 ± 3.11	41.69 ± 3.96	41.11 ± 3.71	0.207	0.078	0.444
T4	41.54 ± 3.62	40.19 ± 5.60	40.43 ± 3.51	0.084	0.252	0.799
T5	41.16 ± 4.00	40.43 ± 4.40	39.86 ± 3.83	0.303	0.141	0.506
T6	41.52 ± 3.79	39.72 ± 5.98	39.75 ± 3.71	**0.035**	0.098	0.978
Prealbumin (mg/L)						
T0	274.79 ± 50.26	273.80 ± 48.70	261.47 ± 56.76	0.910	0.222	0.243
T1	292.37 ± 59.30	284.77 ± 50.34	259.71 ± 69.72	0.443	**0.009**	**0.039**
T2	283.39 ± 61.60	286.49 ± 58.80	253.21 ± 62.84	0.767	**0.020**	**0.009**
T3	270.79 ± 54.71	258.76 ± 54.35	232.38 ± 72.25	0.237	**0.002**	**0.032**
T4	264.13 ± 64.29	241.83 ± 57.52	226.12 ± 65.88	**0.040**	**0.005**	0.223
T5	266.52 ± 62.20	251.33 ± 63.29	226.47 ± 76.63	0.185	**0.005**	0.071
T6	262.30 ± 58.93	245.49 ± 61.15	222.72 ± 66.02	0.129	**0.004**	0.084

Variance analysis was conducted between each group. Bold values means the p value is less than 0.05.

NRS2002 scores increased as treatment continued (**Figure 2C**), and differences became significant between the mild and moderate groups since T3 (p ≤ 0.001; **Table 3**), and between moderate and severe groups since T2 (p = 0.005–0.046). Differences were significant at each time point between the mild and severe groups (p ≤ 0.041).

**Figure 2F** showed that the three subgroups had nearly the same prealbumin levels before RT (T0). However, in the mild group, prealbumin levels increased at T1 and declined subsequently with a slight rise at T5, while it decreased in the severe group since the start of treatment. In the moderate group, prealbumin levels were similar to those of the mild group and decreased at T3-T4, then increased to an intermediate level since T5. There were significant differences in prealbumin levels between the mild and severe groups since T1 (p = 0.002–0.02; **Table 5**), between the mild and moderate groups at T4 (p = 0.040), and between the moderate and severe groups at T1-T3 (p = 0.009–0.039).

Since NRS2002 score and prealbumin levels were significantly different between each subgroup with One Way ANOVA, we further used Repeated Measures ANOVA to verify it, which showed that the differences remained significant (p < 0.001, p = 0.041; **Supplemental Tables 2, 3**).

### Benefits of Nutritional Support

Among three subgroups, there were no significant differences in the proportion of patients receiving general nutritional support and parenteral nutrition (p = 0.055, p = 0.085; **Table 6**). The difference in the proportion of patients receiving enteral nutrition was significant (p = 0.045).

**Table 6:** Comparison between mild, moderate, and severe groups in general nutritional support, enteral nutrition, parenteral nutrition, duration of parenteral nutrition, starting time of enteral and parenteral nutrition, and total calories provided by nutritional support at T0–T6 during chemoradiotherapy (CRT)/radiotherapy (RT) treatment.

Items	Mild group	Moderate group	Severe group	*p*-value
General nutrition support, n/N (%)	42/67 (62.7)	58/75 (77.3)	28/34 (77.3)	0.055
Enteral nutrition, n/N (%)	32/67 (47.8)	44/75 (58.7)	25/34 (73.5)	**0.045**
Parenteral nutrition, n/N (%)	23/67 (34.3)	38/75 (50.7)	18/34 (52.9)	0.085
Duration of parenteral nutrition, mean ± SD (days)	3.06 ± 5.90	5.28 ± 7.38	7.24 ± 10.16	**0.025**
Starting time of enteral nutrition, mean ± SD (week)	2.58 ± 1.67	3.07 ± 1.54	2.92 ± 1.34	0.400
Starting time of parenteral nutrition, mean ± SD (week)	4.34 ± 1.48	3.74 ± 1.33	3.22 ± 1.68	0.055
Total calories provided by nutritional support, mean ± SD (kcal)	7121.91 ± 8471.15	9064.01 ± 9380.13	14860.71 ± 13551.62	**0.001**

Chi-square test and variance analysis were conducted. Bold values means the p value is less than 0.05.

The starting time of enteral nutrition was approximately week 3 in the three subgroups (p = 0.400). Although not statistically different, the starting time of parenteral nutrition was week 4 in the mild and moderate groups, and week 3 in the severe group (p = 0.055). The duration of parenteral nutrition was the longest in the severe group, and the total calories provided by nutritional support increased as ROM severity worsened (p = 0.025; p = 0.001).

## Discussion

Malnutrition is a common problem in NPC patients during RT as a result of gastrointestinal reactions to concurrent chemotherapy, xerostomia, psychological distress, and ROM (21–24). Severe weight loss and poor physical condition due to malnutrition may lead to CRT interruptions, poor treatment tolerance, and abandonment of concurrent chemotherapy, which eventually impact prognosis (25, 26). Numerous trials have reported that certain nutritional factors are correlated with disease survival outcomes and distant metastasis in various malignancies including NPC, which highlight the significance of adequate nutritional status in NPC patients during treatment (27–33).

Previous studies have reported the deteriorating nutritional status of NPC patients during RT (15, 16, 34–39). However, most studies only reported the weight loss after treatment, and nutritional status was evaluated only before and at the end of RT, as opposed to during treatment. In a prospective study, the median weight loss during RT was 6.9 kg (2.1–12.6 kg), representing 3.5% to 16.4% weight loss (36). Jager-Wittenaar reported the average weight loss was 3.6 kg, which was 4.7% of pre-treatment body weight (38). In the Nourissat’s study, even though the average weight loss decreased to 2.2 kg with only 25% of HNC patients reporting severe weight loss (≥ 5%), researchers concluded that the rate was likely underestimated (40). In our study, mean weight loss was 6% of pre-treatment body weight at the end of RT, and we further found body weight and BMI significantly decreased since T1 and continued decreasing throughout the entire RT process, and the percentage of average weight loss was already 5% at T5. Actually, weight loss had already started prior to RT (36, 41, 42), revealing the presence of malnutrition before RT was initiated. The reason may be attributed to metabolic and endocrine changes and hypercatabolism caused by the responsiveness to chemotherapy, which make malnutrition cannot be fully reversed by conventional nutritional support (43). In addition, body weight is not a sensitive nutrition parameter over a short period of time (44, 45). Like body weight and BMI, the change of NRS2002 score also revealed the deteriorating nutritional status of patients, which further supported the conclusion that it was quite necessary to provide nutritional support and education at the start of RT.

Albumin is commonly used as a nutritional marker of protein-energy in clinical practice, however, we found prealbumin that responded quickly to nutritional interventions was more sensitive and suitable for NPC patients (46–48). Although a significant decrease in prealbumin levels was observed after RT compared with baseline like previous studies (15, 39), we found an increase at T1 and T5. Since all patients received nutrition education before treatment and the nutritional support had become more frequently in our cancer center, we speculated that prealbumin levels increased at T1 due to early nutrition education and at T5 due to nutritional support and re-education, which again proved the importance of early nutritional support.

The association between nutritional status and ROM was investigated. To our knowledge, this is the first prospective observational study that specifically worked on this. We innovatively divided patients into three subgroups: mild, moderate and severe groups. The results of NRS2002 scores and prealbumin levels showed that there was a strong association between nutritional status and ROM, indicating that malnutrition was largely caused by ROM. What beyond our expectation is that we found malnutrition was not only the consequence of ROM. As presented in **Table 3**, the difference between mild and severe group in NRS2002 score at T0 was significant, which indicating that malnutrition was very likely to be a risk factor to ROM. Although not significant, the prealbumin level of mild and moderate groups was higher than severe group at T0 as well. Our previous study also found that body weight loss ≥ 5% was a related risk factor to severe ROM (19). Two other studies conducted in oral cavity cancer patients found that lower BMI was significantly related with severe ROM (49, 50). The underlying mechanism might be that malnutrition could interfere with mucosal regeneration due to decreased cellular migration and renewal resulting from poor nutrition status (51). In conclusion, nutritional support should be more frequent and earlier not only to reverse malnutrition due to ROM but also reduce the risk of developing severe ROM.

We further compared nutritional support among the three subgroups, and found significant differences in the proportion of patients receiving enteral nutrition, duration of parenteral nutrition, and total calories provided by nutritional support. However, the severe group still had the worst nutritional status, which meant that nutritional support should be provided earlier than usual.

The study had several limitations. Although the sample size was not small, we excluded 96 patients mainly due to lack of comprehensive data (prealbumin and albumin test, data on nutritional support, etc), which might cause bias. As mentioned above, there were no standard guidelines or procedures for nutritional support, the conclusion of differences in nutritional support among three subgroups was not that reliable.

## Conclusion

Malnutrition is very common in NPC patients and occurs earlier than usually expected during RT. ROM is strongly associated with nutritional status, which might be bidirectional. Therefore, adequate nutritional support should be provided to all NPC patients at the start of RT, especially those at high-risk of severe ROM. Thus, further studies are needed to explore approaches to identify high-risk patients.

## Data Availability Statement

The data analyzed in this study is subject to the following licenses/restrictions: this paper was generated from a part of the datasets, which will be used in other papers that focused on different research topic. Thus, the datasets were not publicly available. Requests to access these datasets should be directed to chenyy@zjcc.org.cn.

## Ethics Statement

The study was approved by five medical centers, Zhejiang Cancer Hospital (IRB-2018-180), Sun Yat-sen University Cancer Center (B2019-069-01), Affiliated Cancer Hospital of Zhengzhou University (2019268), Affiliated Hospital of Guangdong Medical University (PJ2019-063), and The Affiliated Huaian No.1 People’s Hospital of Nanjing Medical University (YX-P-2019-059-01). The patients provided their written informed consent to participate in this study.

## Author Contributions

YC and LL designed the research. ZS, ZZ, and BY disposal data. JC and WS performed the statistical analysis; Other authors made efforts to recruit patients. All authors contributed to the article and approved the submitted version.

## Funding

The study received financial support from Qilu Pharmaceutical Co., Ltd.

## Conflict of Interest

Authors JC and WS were employed by the company Hangzhou YITU Healthcare Technology Co., Ltd.

The remaining authors declare that the research was conducted in the absence of any commercial or financial relationships that could be construed as a potential conflict of interest.

The authors declare that this study received funding from Qilu Pharmaceutical Co., Ltd. The funder was not involved in the study design, collection, analysis, interpretation of data, the writing of this article or the decision to submit it for publication.
