# Supplementary material for: Nutritional Status and Its Association With Radiation-Induced Oral Mucositis in Patients With Nasopharyngeal Carcinoma During Radiotherapy: A Prospective Study
Source: Front Oncol. 2020 Nov 6;10:594687. doi: 10.3389/fonc.2020.594687 (PMC7677572; doi:10.3389/fonc.2020.594687)
Supplement: Supplementary file 1 [file DataSheet_1.docx]

Supplemental Table 1. CTCAE grade for oral mucositis

| Grade 1 | Asymptomatic or mild symptoms; intervention not indicated |
| --- | --- |
| Grade 2 | Moderate pain; not interfering with oral intake; modified diet indicated |
| Grade 3 | Severe pain; interfering with oral intake |
| Grade 4 | Life-threatening consequences; urgent intervention indicated |
| Grade 5 | Death |
| Note: Grade 0 is not applied. All patients with no symptoms or signs of oral mucositis is classified into Grade 1. | |

Supplemental Table 2. Repeated Measures ANOVA for NRS2002 score between three subgroups

| Sourse | Type III Sum of Squares | df | Mean Square | | F | p |
| --- | --- | --- | --- | --- | --- | --- |
| Intercept | 4966.868 | 1 | 4966.868 | 1568.322 | | **< 0.001** |
| Groups | 121.151 | 2 | 60.576 | 19.127 | | **< 0.001** |
| Error | 544.723 | 172 | 3.167 |  | |  |

Supplemental Table 3. Repeated Measures ANOVA for prealbumin levels between three subgroups

| Sourse | Type III Sum of Squares | df | Mean Square | | F | p |
| --- | --- | --- | --- | --- | --- | --- |
| Intercept | 65490009.8 | 1 | 65490009.8 | 3720.41 | | **< 0.001** |
| Groups | 114556.890 | 2 | 57278.445 | 3.254 | | **0.041** |
| Error | 2675906.28 | 152 | 17604.647 |  | |  |
